# Analysis of resveratrol as a lung cancer chemopreventive agent in A/J mice exposed to benzo[*a*]pyrene

**DOI:** 10.1038/sj.bjc.6602125

**Published:** 2004-08-17

**Authors:** G Berge, S Øvrebø, E Eilertsen, A Haugen, S Mollerup

**Affiliations:** 1 Department of Toxicology, National Institute of Occupational Health, PO Box 8149 Dep, N-0033 Oslo, Norway

**Keywords:** lung carcinogenesis, benzo[*a*]pyrene, resveratrol, adducts, CYP1A1, CYP1B1

## Abstract

Resveratrol inhibits PAH bioactivation through reduced expression of the CYP1A1 and CYP1B1 genes in human bronchial epithelial cells. *Ad libitum* access to a diet containing resveratrol showed no effect on benzo[*a*]pyrene-induced lung tumorigenesis in A/J mice. Also, resveratrol did not change CYP1A1 and CYP1B1 gene expression or benzo[*a*]pyrene protein adduct levels in the lung tissue. The lack of chemopreventive activity may have been caused by insufficient concentrations or nonreactive forms of resveratrol in the lungs.

Lung cancer is the major cause of cancer-related mortality worldwide and tobacco smoke is established as the primary aetiologic factor for the disease. Other risk factors are occupational exposure and urban air pollution (Twombly, 2003). Today, 25–30% of adults in western populations are active smokers, while the number is increasing in developing countries (Peto *et al*, 1996). The cancer has proven difficult to control with conventional therapeutic and surgical approaches, and the prognosis is poor with an overall 5-year survival rate of 10–14% in the USA (Jemal *et al*, 2004). The use of naturally occurring or synthetic agents to prevent, inhibit or reverse lung carcinogenesis would therefore greatly benefit public health. Resveratrol (trans-3,4′,5-trihydroxystilbene) is a phenolic phytoalexin present in wines, berries and nuts, which has shown chemopreventive potential (Jang *et al*, 1997).

Benzo[*a*]pyrene (B[*a*]P) is a major carcinogenic constituent in tobacco smoke (Hecht, 2003). It is metabolically activated by the cytochrome *P*450 (CYP) system to reactive diolepoxides which are capable of interacting with DNA or proteins to form adducts. In the lung, CYP1A1 and CYP1B1 are important in the biotransformation of B[*a*]P, and their expression is inhibited by resveratrol *in vitro* (Ciolino and Yeh, 1999; Mollerup *et al*, 2001; Berge *et al*, 2004b). Accordingly, in human bronchial epithelial cells, inhibition of CYP1A1 and CYP1B1 was accompanied by reduced formation of the ultimate carcinogen BPDE-I and BPDE–DNA adducts (Mollerup *et al*, 2001; Berge *et al*, 2004b).

The anticancer effect of resveratrol has previously been studied with conflicting results *in vivo*. In this study, we addressed the effect of resveratrol on initiation of lung tumorigenesis in A/J mice. Mice with free access to a diet containing resveratrol were repeatedly exposed to B[*a*]P by gavage. The effect of resveratrol on the expression level of CYP1A1 and CYP1B1 in the lung tissue was determined by quantitative real-time RT–PCR, and hydrolysed B[*a*]P–protein adducts were measured by HPLC. Furthermore, the development of lung tumours in response to resveratrol was investigated.

## MATERIALS AND METHODS

### Animal handling and treatment

A total of 150 female A/JOlaHsd mice (Harlan, UK) were housed in an animal facility with a 12-h light/dark cycle at 21°C and 55% RH. The mice had *ad libitum* access to tap water and diet throughout the study. Diet was prepared daily by dispensing EtOH only (solvent control) or resveratrol/EtOH to a pulverised standard diet (RM1)(SDS, UK) (0.4% w w^−1^), and the solvent was evaporated overnight in the dark. Food dishes were replaced each morning and were shaded by metal plates over the cages. The stability of trans-resveratrol in the diet was tested by HPLC, and no decomposition was found. The mice consumed 5–6 g day^−1^, resulting in 6–8 mg kg^−1^ resveratrol ingested. The administered dose of resveratrol was chosen based on analyses described in the literature (Kimura and Okuda, 2001; Asensi *et al*, 2002). After 1 week on the diet, the B[*a*]P exposure regimes were started on 6-week-old animals. B[*a*]P (in corn oil) was delivered by a gastric tube (i.g.) once a week for 8 weeks, giving a total dose of 80 or 300 mg kg^−1^. Control animals received corn oil only. There were no differences in the food intake, weight increase, or behaviour between the groups, and no indications of toxicity of resveratrol or B[*a*]P (data not shown). The animals were randomly divided into six groups (*n*=25). At 24 h after the last dose of B[*a*]P, five animals in each group were killed by cervical dislocation. The lungs and livers were excised, snapfrozen in liquid nitrogen, and stored at −70°C. At 5 months after the last dose of B[*a*]P, the rest of the animals were killed. The lungs were fixed in Bouin's fluid. The tracheae were removed and the individual lobes were dissected free and transferred to 96% ethanol. The number of tumours was determined by manual counting and the size scored according to a ruler in the microscope. Lung tumour development was as expected for the strain, doses and exposure regimen of B[*a*]P (Hecht *et al*, 1994). *In vivo* testing was performed according to Workman *et al* (1998), and all animal-handling and experimental procedures were conducted in conformity with the laws and regulations controlling experiments on live animals in Norway and the European Convention for the Protection of Vertebrate Animals used in Experimental and Other Scientific Purposes.

### Real-time RT–PCR

Total RNA was extracted from tissue and mRNA was reverse transcribed using random primers as described previously (Berge *et al*, 2004b). Sequences for the PCR primers were: CYP1A1 forward, 5′-ACC TTC CGG CAT TCA TCC TT-3′; CYP1A1 reverse, 5′-GCC ATT CAG ACT TGT ATC TCT TGT G-3′; CYP1B1 forward, 5′-GTG GCT GCT CAT CCT CTT TAC C-3′; CYP1B1 reverse, 5′-CCC ACA ACC TGG TCC AAC TC-3′; *β*-actin forward, 5′-GAC AGC ACA GCC TGG ATG GCT A-3′; *β*-actin reverse, 5′-GTG AAA AGA TGA CCC AGA TCA-3′. Real-time PCR was performed on an ABI PRISM 5700 (Applied Biosystems, Foster City, CA, USA) with SYBRgreen I (40 cycles of 95°C 15 s^−1^, 60°C 1 min^−1^). The amount of target cDNA in each sample was established by determining a fractional PCR threshold cycle number (Ct). The relative expression of each gene normalised to *β*-actin was calculated as 2^−ΔCt^, where ΔCt=Ct_gene_−Ct_*β*-actin_.

### B[*a*]P–protein adduct measurements

B[*a*]P–protein adducts were measured as released B[*a*]P-tetrol after acid hydrolysis. The preparation of the tissue and purification by Sep-Pak C_18_ cartridge (Millipore Corporation, Milford, MA, USA) was performed as described in Berge *et al* (2004a). High-performance liquid chromatography (HPLC) separation of the B[*a*]P-tetrols was performed on a Hypercil C_18_ column 4.6 × 150 mm and 5 *μ*m (Agilent Technologies, Waldbronn, Germany) in a linear gradient of 30–100% methanol for 40 min on an Agilent 1100 system. The following fluorescence conditions were used: 0 min, ex 341/em 381; 20 min, ex 253/em 410; 27 min, ex 380/em 431. The concentrations of B[*a*]P metabolites were determined by comparison to standards from the NIH Chemical Carcinogen Repository (Midwest Research Institute, Kansas City, MO, USA).

### Tissue distribution of resveratrol

Lung tissue, intestinal tissue (small intestines and colonic mucosa combined) and faeces were collected. The tissue samples were homogenised by a polytron in NaAc (0.1 M, pH 5.0), and further incubated with *β*-glucoronidase and arylsulphatase (Roche) at 37°C overnight to cleave the sulphate and glucuronide conjugates of resveratrol. The samples were sonicated three times in EtOH, centrifuged and the precipitates were washed with EtOH, evaporated to dryness, dissolved in 3 ml H_2_O and purified on Sep-Pak cartridges (based on Vitrac *et al*, 2003). Resveratrol was quantified by HPLC in a linear gradient of 10–100% methanol using a diode array detector (DAD) at 306 nm. The detection limit of resveratrol by HPLC was approximately 1 pmol (signal to noise=3).

### Statistical analyses

For the analysis of gene expression, protein adducts and tumour size, means were compared by the independent-samples *t*-test. With significant variations in standard deviation, Welch correction was applied. Variations in the number of tumours were investigated by Fisher's exact test and *χ*^2^ test (SPSS, Chicago, IL, USA).

## RESULTS

**Figure 1:**
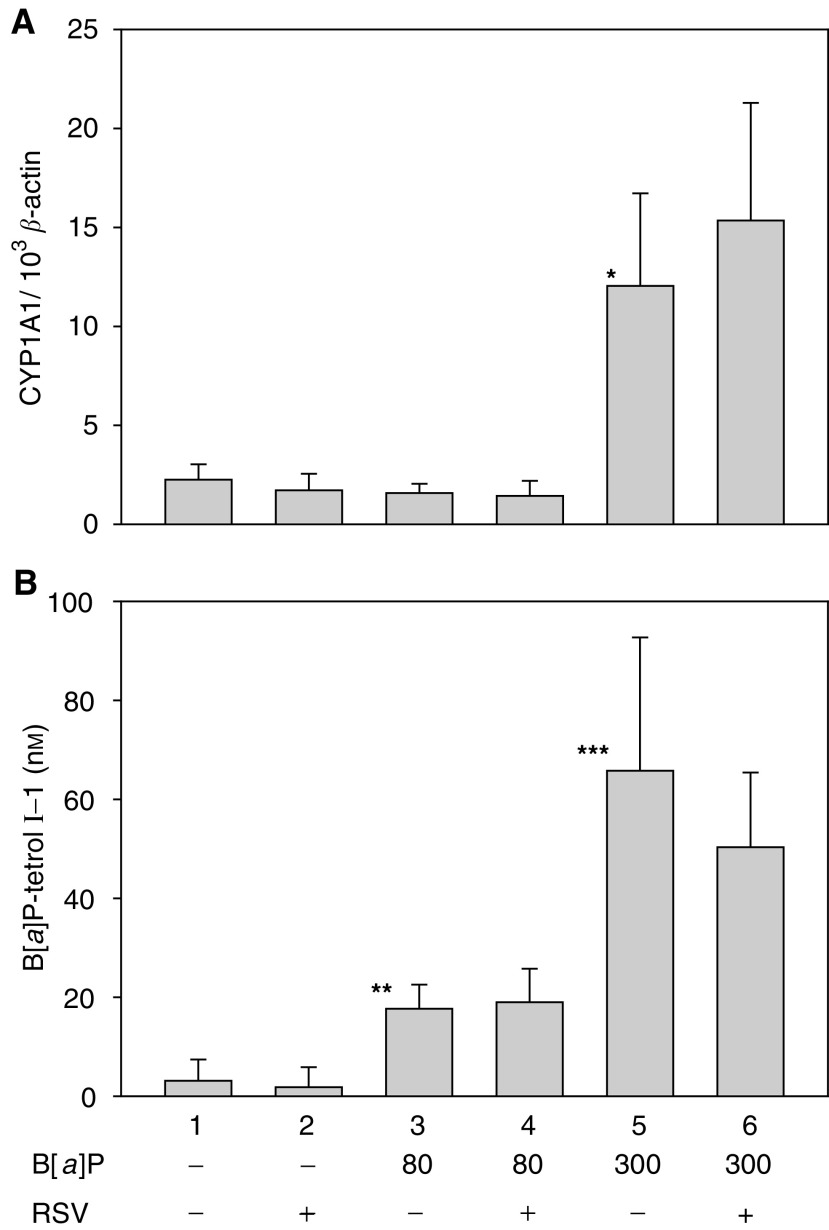
The mice were orally injected with corn oil or B[*a*]P (mg kg^−1^ cumulative dose), and received resveratrol (RSV) in the diet as indicated. They were killed 24 h after the eight dose of B[*a*]P. (**A**) Real-time RT–PCR measurement of CYP1A1 expression relative to the expression of *β*-actin in lung tissue. Expression levels were normalised to *β*-actin. Columns and error bars represent the mean and s.d. (*n*=5). ^*^Lane 1 *vs* lane 5, *P*=0.01, independent samples *t*-test with Welch correction. (**B**) Effect of resveratrol on B[*a*]P–protein adduct formation in lung tissue. The levels of the protein-adduct hydrolysis product B[*a*]P-tetrol I-1 were measured by fluorescence HPLC. Columns and error bars represent the mean and s.d. (*n*=5). ^**^Lane 1 *vs* lane 3, *P*=0.0001 independent samples *t*-test. ^***^Lane 1 *vs* lane 5, *P*=0.004 independent samples *t*-test with Welch correction.

CYP1A1 and CYP1B1 gene expression was measured in the lungs of mice given a weekly dose of B[*a*]P for 8 consecutive weeks. The expression level of CYP1A1 was found to be low in the control and low-dose B[*a*]P groups, whereas a dose of 300 mg kg^−1^ B[*a*]P significantly induced the gene (Figure 1A

). The addition of resveratrol in the diet did not significantly affect the expression levels of CYP1A1 in any group (solvent only, 80 mg kg^−1^ B[*a*]P, or 300 mg kg^−1^ B[*a*]P) compared to the respective controls. B[*a*]P was not found to induce significant changes in the mRNA level of CYP1B1, while the basal expression level of the gene was higher than for CYP1A1. Resveratrol did not alter the expression level of CYP1B1 significantly, regardless of B[*a*]P exposure dose (data not shown).

The hydrolysis of protein adducts resulted in various amounts of B[*a*]P-tetrols, of which B[*a*]P-tetrol I-1 (a derivate of the B[*a*]P-oxide BPDE-I) was the most abundant. The levels of B[*a*]P-tetrol I-1 increased dose-dependently in response to B[*a*]P (Figure 1B). No significant effect of resveratrol was found on the level of B[*a*]P-tetrol I-1 protein adducts at either low or high dose of B[*a*]P, compared to the corresponding controls. The results for the other B[*a*]P hydrolysis products showed similar trends or were below detection limits (data not shown). B[*a*]P–protein adducts were also measured in liver tissue from the mice. As for the lung, no effect of resveratrol was found (data not shown).

The remaining animals were killed 5 months after the last dose of B[*a*]P and lung tumours were examined. The number of tumours increased dose-dependently with B[*a*]P exposure, while no significant effect of resveratrol was found (Table 1

**Table 1:** Effect of resveratrol on development of tumours in mouse lung tissue

**Compound**	**Solvent**	**RSV**	**80 mg kg^−1^ B[*a*]P**	**80 mg kg^−1^ B[*a*]P+RSV**	**300 mg kg^−1^ B[*a*]P**	**300 mg kg^−1^ B[*a*]P RSV**
Mice with tumour	7/20	7/20	16/20	15/20	20/20	20/20
Tumours/mice	0.35	0.35	1.6	1.35	14.45	15.4
Size±s.d. (mm)	0.63	0.70	0.45±0.20	0.47±0.21	0.54±0.17	0.53±0.18

). Similarly, resveratrol was not found to significantly alter the size of the tumours when the respective groups were compared.

By HPLC, no resveratrol or resveratrol conjugates were found in the lung tissue of animals after dietary administration of the compound. However, resveratrol could be detected in intestinal tissue (89.9 pmol g^−1^ tissue) and faeces (9.6 nmol g^−1^ faeces).

## DISCUSSION

We have previously shown that resveratrol inhibits CYP1A1 and CYP1B1 expression, as well as the formation of B[*a*]P–protein and –DNA adducts in cultured human bronchial epithelial cells (Mollerup *et al*, 2001; Berge *et al*, 2004b). To explore the lung cancer chemopreventive potential of resveratrol in a physiologically relevant *in vivo* scenario, the compound was administered orally to A/J mice. This strain is frequently used as an experimental model of lung carcinogenesis, and is well suited as the mice readily produce lung tumours after oral B[*a*]P exposure (Hecht *et al*, 1994). In short, the results of our study under these conditions are not indicative of an effect of resveratrol on either the number or size of B[*a*]P-induced lung tumours in A/J mice.

The effect of resveratrol on the development of different cancer types has previously been assayed in several *in vivo* studies with various results. Our data resemble the findings of Hecht *et al* (1999), who did not observe any effect on lung tumour multiplicity when resveratrol was given in the diet to A/J mice from 1 week after oral carcinogen exposure. To study the effect during initiation, however, resveratrol was administered from 1 week before the start of B[*a*]P exposure and continued throughout the study period. Resveratrol was not found to significantly alter the expression levels of CYP1A1 or CYP1B1 or the B[*a*]P protein–adduct level in lung, contrasting our *in vitro* data (Mollerup *et al*, 2001; Berge *et al*, 2004b). Few studies have quantitatively addressed the effect of resveratrol in intact lung tissue. In one study, subcutaneous injection of resveratrol was by semiquantitative immunohistochemistry or Western blotting found to inhibit the level of B[*a*]P-induced CYP1A1 in mouse lung tissue (Revel *et al*, 2003).

The efficacy of orally administered resveratrol will, in addition to the mechanism of action, depend on its absorption, metabolism, and pharmacokinetic tissue distribution. To resemble a relevant physiological condition, we chose to deliver resveratrol in the diet, as oral administration would be a preferable route in cancer chemoprevention. Also, repeated intake could result in an elevated basal level of the compound in the plasma (Scalbert and Williamson, 2000; Asensi *et al*, 2002). By autoradiography and HPLC, intact trans-resveratrol has been detected in mouse lung tissue after a single intragastric delivery of various doses (Vitrac *et al*, 2003; Sale *et al*, 2004). On the other hand, recent studies have suggested rapid clearance in mice tissue after a single oral dose of resveratrol (Asensi *et al*, 2002; Yu *et al*, 2002). While both resveratrol and its conjugates have been detected in the small intestine and colonic mucosa, the full pharmacologic distribution is not known (Vitrac *et al*, 2003; Sale *et al*, 2004). Due to the lack of *in vivo* effect in the present study, we investigated the bioavailability of resveratrol in the mouse tissue. No resveratrol or resveratrol conjugates were found by HPLC in the lung tissue of animals receiving the compound in the diet.

Resveratrol is known to undergo metabolic phase II reactions involving conjugation with sulphate and glucuronic acid, which may influence the biological effect of the compound. The extent of such modification in various tissues is not known (Scalbert and Williamson, 2000; Yu *et al*, 2002). Resveratrol has shown anticarcinogenic effects in colon, but the data are conflicting. In Min mice, a strain predisposed to develop intestinal tumours, resveratrol administered in the drinking water strongly reduced the formation of colon and small intestinal tumours (Schneider *et al*, 2001). However, the doses used in this study have been questioned by Ziegler *et al* (2004), who found no effect of resveratrol in the diet on either COX-2 expression or the number of tumours. In the negative studies, including the present, resveratrol given in the diet may not have reached the target tissue in sufficient concentrations or biological active form. However, the potential beneficial effects on health justify further studies of the absorption, metabolism and disposition of trans-resveratrol in the tissue in question.

Inhibition of CYP-gene expression accompanied by a reduced formation of carcinogenic B[*a*]P metabolites and DNA adducts may be an important step in preventing or lowering the risk of lung cancer. Data concerning effects of resveratrol on the prevention of cancer are ambiguous and lack a link between target organ, efficacy *in vivo* and the activity observed *in vitro* (Gescher and Steward, 2003). The current data, based on oral administration, do not lend support for resveratrol as a physiologically effective chemopreventive agent for the inhibition of PAH-induced lung cancer.
